# Interventions for Digital Addiction: Umbrella Review of Meta-Analyses

**DOI:** 10.2196/59656

**Published:** 2025-02-11

**Authors:** Peng Lu, Jiamin Qiu, Shiqi Huang, Xinman Wang, Shasha Han, Sui Zhu, Youjing Ning, Fang-fang Zeng, Yuan Yuan

**Affiliations:** 1 Department of Chinese International Education Chinese Language and Culture College Huaqiao University Xiamen China; 2 Department of Public Health and Preventive Medicine School of Medicine Jinan University Guangzhou China; 3 Department of Neonatology and Pediatrics The First Affiliated Hospital of Jinan University Jinan University Guangzhou China

**Keywords:** digital addiction, interventions, umbrella reviews, systematic review, internet addiction, loneliness, quality of life, well-being, internet, psychological, pharmacological treatment, cognitive behavioral therapy

## Abstract

**Background:**

Numerous studies have explored interventions to reduce digital addiction outcomes, but inconclusive evidence makes it difficult for decision-makers, managers, and clinicians to become familiar with all available literature and find appropriate interventions.

**Objective:**

This study aims to summarize and assess the certainty of evidence of interventions proposed to decrease digital addiction from published meta-analyses.

**Methods:**

An umbrella review of published meta-analyses was performed. We searched PubMed, Cochrane Library, Web of Science, and Embase for meta-analyses published up to February 2024. Eligible studies evaluated interventions using randomized controlled trials, nonrandomized controlled trials, or quasi-experimental studies and were assessed for methodological quality using Assessment of Multiple Systematic Reviews version 2. A random effects model was used to analyze data, considering heterogeneity and publication bias. Grading of Recommendations, Assessment, Development, and Evaluations was applied to assess evidence with certainty.

**Results:**

A total of 5 studies assessing 21 associations were included in the umbrella review, of which 4 (80%) were high-quality meta-analyses. Weak evidence was observed in 19 associations, whereas null associations appeared in the remaining 2 associations. These associations pertained to 8 interventions (group counseling, integrated internet addiction [IA] prevention programs, psychosocial interventions, reality therapy, self-control training programs, cognitive behavioral therapy, interventions to reduce screen time in children, and exercise) and 9 outcomes (self-control, self-esteem, internet gaming disorder symptoms, time spent gaming, IA scores, screen use time, interpersonal sensitivity longlines, anxiety, and depression). Cognitive behavioral therapy reduces anxiety (standardized mean difference [SMD] 0.939, 95% CI 0.311 to 1.586), internet gaming disorder symptoms (SMD 1.394, 95% CI 0.664 to 2.214), time spent gaming (SMD 1.259, 95% CI, 0.311 to 2.206), and IA scores (SMD –2.097, 95% CI –2.814 to –1.381). Group counseling had a large effect on improving self-control (SMD 1.296, 95% CI 0.269 to 2.322) and reducing IA levels (SMD –1.417, 95% CI –1.836 to –0.997). Exercise intervention reduced IA scores (SMD –2.322, 95% CI –3.212 to –1.431), depression scores (SMD –1.421, 95% CI –2.046 to –797), and interpersonal sensitivity scores (SMD –1.433, 95% CI –2.239 to –0.627).

**Conclusions:**

The evidence indicates that current interventions to reduce digital addiction are weak. Data from more and better-designed studies with larger sample sizes are needed to establish robust evidence.

**Trial Registration:**

PROSPERO CRD42024528173; crd.york.ac.uk/PROSPERO/display_record.php?RecordID=528173

## Introduction

The World Health Organization has officially recognized digital addiction as a global problem in which excessive digital activities and internet use lead to the inability to manage time, decreased energy and concentration during the day, and procrastination of bedtime and shortened total sleep time due to disrupted sleep patterns at night, thereby reducing an individual’s subjective happiness [1,2]. A survey in 31 countries revealed that 6% of internet users aged 12-41 years had symptoms of digital addiction [3]. The prevalence of digital addiction varies globally, with an average of 4.6% in Western countries and 8.9% in other countries [4]. Digital addiction has negative effects on personal work and life; damages personal physical and mental health; causes physical health problems such as fatigue and a lack of sleep; causes negative emotions such as anxiety, depression, and anger; reduces work concentration and work efficiency; damages social relationships; leads to loneliness; reduces life satisfaction; and affects the growth of adolescents [5-8].

Many studies in the fields of psychology and medicine have focused on the harms of digital addiction and potential intervention strategies. Moreover, numerous studies have pointed to personal traits and emotional self-regulatory mechanisms (eg, self-esteem and self-control) as key factors associated with the propensity to become digitally addicted [9-11]. In addition, elevated “fear of missing out” awareness compels individuals to consistently use digital networks to maintain social connections, which may lead to inappropriate use of digital media and the emergence of anxiety symptoms that exacerbate digital addiction [12,13]. Resilience is an expression of positive adaptation in the face of great adversity, which can neutralize the harmful effects of stress and thus reduce the tendency to become addicted to the internet; however, when faced with stress that exceeds their resilience, individuals may be inclined to overuse the internet as a coping strategy to alleviate stress [14,15]. Owing to the magnitude of the impact of psychological disorders on digital addiction, for individuals, the prevention and treatment of digital addiction are mainly psychological and pharmacological [1]. Beute et al [16] report that cognitive behavioral therapy (CBT) can play a role in treating adolescent internet addiction (IA)**.** CBT and CBT-based psychotherapies are the psychotherapy most commonly used for digital addiction [17]. Similarly, psychosocial intervention, a unique type of psychotherapy, is believed to alleviate digital addiction by enhancing an individual's self-esteem and self-regulation [18]. In addition, adjuvant therapies such as sports interventions can be effective in preventing and treating IA [19]. Alternative therapy uses alternative methods such as reading to reduce dependence on the internet, thereby achieving the purpose of relieving emotions. Many researchers have also tried to combine two or more interventions to achieve better therapeutic effects.

Currently, there are several meta-analyses on the prevention and intervention of digital addiction [20-22]. Liu et al [20] combine 58 randomized controlled trials (RCTs) and report that group counseling programs, CBT, and sports interventions reduce IA symptoms. Matthew et al [22] report that CBT was a short-term intervention for reducing internet gaming disorder (IGD) and depression symptoms, but its role in reducing actual time spent gaming was unknown. A study by Zhang et al [21] indicates that exercise can effectively reduce the symptoms of anxiety, depression, and loneliness among internet-addicted students and is beneficial to mental health. However, the heterogeneity of most meta-analyses on the treatment and prevention of digital addiction, due to reasons such as lack of a consistent definition, different outcome measurements, and wide variation in study design, limit the clinical impact of such results. Therefore, it is necessary to summarize the current evidence related to digital addiction intervention methods and conduct an overall assessment of their quality based on comprehensive evidence grading standards by using umbrella reviews which could consolidate the highest quality level of evidence by merging and assessing the meta-analyses [23,24].

Accordingly, this study conducted an umbrella analytic review of relevant meta-analyses with the purpose of evaluating the effects of various intervention modalities for digital addiction; systematically examined, organized, and assessed the strength of evidence and potential degree of bias from multiple reviews and meta-analyses; and conducted a literature review of interventions for digital addiction to identify the most effective interventions and provide recommendations to guide future health-related research and develop effective therapeutic interventions.

## Methods

### Study Design

This umbrella review was conducted in accordance with the registered protocol (Open Science Framework Registry) and the Preferred Reporting Items for Systematic Reviews and Meta-Analyses (PRISMA) 2020 statement [25]. Our study protocol has been registered with PROSPERO.

### Search Strategy and Selection Criteria

We searched the following databases for English language studies published up to February 2024: PubMed, the Cochrane Library, Web of Science, and Embase. We used key terms related to digital addiction and interventions. The following search strategy was used: (“Internet” OR “digital” OR “screen” OR “net” OR “online” OR “media” OR “electronic device” OR “electronic gadgets” OR “computer” OR “mobile” OR “phone” OR “smartphone” OR “television” OR “TV” OR “video” OR “Facebook” OR “game” OR “gaming”) AND (“addict” OR “use” OR “dependent” OR “overuse” OR “abuse” OR “disorder” OR “excessive” OR “effects” OR “habits” OR “intervene” OR “treatment” OR “therapy” OR “compulsive” OR “heavy”) AND (“meta-analy” OR “meta-analytic” OR “metaanaly” OR “meta-regression” OR “meta synthesis” OR “meta-analysis” OR “meta-analysis”) AND (“intervention” OR “interfere” OR “intervene” OR “treatment” OR “therapy” OR “cure” OR “therapeutic” OR “exercise therapy” OR “counseling”). Studies were published in full peer-reviewed literature without language or date restrictions.

The studies included in the meta-analysis had to meet the following inclusion criteria: (1) research topics related to digital addiction intervention; (2) participants diagnosed with digital addiction; (3) a study design limited to RCTs, nonrandomized controlled trials, and quasi-experimental studies; and (4) different forms of digital addiction with similar effects.

The exclusion criteria were as follows: (1) duplicate publications; (2) full-text documents could not be obtained, and other experimental-related basic research was performed; and (3) meta-analysis without any effect size. When 2 or more meta-analyses were performed for the same association, only the most recent systematic review with the largest number of individual studies was included to avoid duplication of samples (Multimedia Appendix 1 [18,20,21,26-39]).

### Study Selection

Documentation records were extracted and imported into Covidence (Veritas Health Innovation). After excluding duplicates, titles and abstracts were assessed independently by 2 researchers. The full texts of the relevant studies were independently reviewed by 2 researchers to ensure that the inclusion and exclusion criteria were met. In the event of discrepancies, a final assessment was made by a third independent researcher.

### Data Extraction and Methodological Quality Assessment

Data extraction was independently completed by 2 researchers. The information extracted from each meta-analysis was as follows: first author; publication year; design and number of included studies; statistical estimates of the meta-analysis; number of original studies; sample size and region; intervention; comparison; any relevant subgroup analyses; and intervention-related outcome indicators. Any disputes during data entry were resolved through discussion with a third researcher. When a meta-analysis contained multiple intervention outcome indicators, each indicator was extracted and analyzed separately.

Two reviewers independently assessed the methodological quality of each systematic review via the Assessment of Multiple Systematic Reviews version 2 (AMSTAR 2) rating scale [40]. AMSTAR 2 consists of 16 items and is a measurement tool used to evaluate meta-analyses. The scale has good consistency, reliability, construct validity, and feasibility. It covers key questions of the included studies, proposals, literature searches, literature screening, data extraction, basic characteristics of the included original studies, data analysis, and conflicts of interest. The overall quality of a systematic review is informed by assessing how well the study meets each of the criteria. Overall quality is classified as “high,” “medium,” “low,” or “very low” [40].

### Data Analysis

The umbrella review compiles the evidence of multiple clinical-related issues in the system reviews and meta-analyses and then comprehensively evaluates the strength of the meta-analyses related to specific issues. First, the combined effect values and their 95% CIs associated with digital addiction interventions in each meta-analysis and their 95% CIs were extracted*,* and the combined effect sizes, 95% CIs, and *P* values were reestimated after the normalization of the mean difference (MD) and Hedges *g* to the standardized mean difference (SMD) via a random effects model (DerSimonian and Laird) by referring to the analyses of the previous omnibus review. *I*^2^ and Cochran *Q* were used to evaluate the heterogeneity of the evidence in the literature, with *I*^2^≥50% or Cochran *Q* test *P* value of less than .10 indicating significant heterogeneity in the evidence. The estimated values of the publication bias of various studies through Egger inspection and evaluation, *P* value of less than .10, indicate that there is a significant publication bias. When the results indicate that there is a significant bias, if the total estimated effect value of the meta-analysis is greater than the effect value of the original research with the largest proportion of its effect, it indicates that the meta-analysis has a small-scale research effect. By determining whether the number of statistical results (*P* value of less than .05) in each meta-analysis (*P* value of less than .05) is greater than its expected quantity (E), the meta-analysis is excessive E, and *P* value of less than .10 indicates excessive significance. The rest of the analyses used a difference of *P* value of less than .05 to determine whether the difference was statistically significant.

The results are presented in forest plots and tables, and all analyses and mapping were performed via Stata (version 17.7, StataCorp) and R (version 4.2.1; R Foundation for Statistical Computing).

### Determining the Credibility of Evidence

The Grading of Recommendations, Assessments, Development, and Evaluations (GRADE) approach was used to evaluate the strength of the evidence in each included study. According to the statistical analysis results, the credibility of each piece of evidence is further divided into 5 levels: I, II, III, IV, and NS, which represent “convincing evidence,” “highly suggestive evidence,” “suggestive evidence,” “weak evidence,” and “insignificant evidence,” respectively [41].

## Results

### Study Characteristics

**Figure 1:**
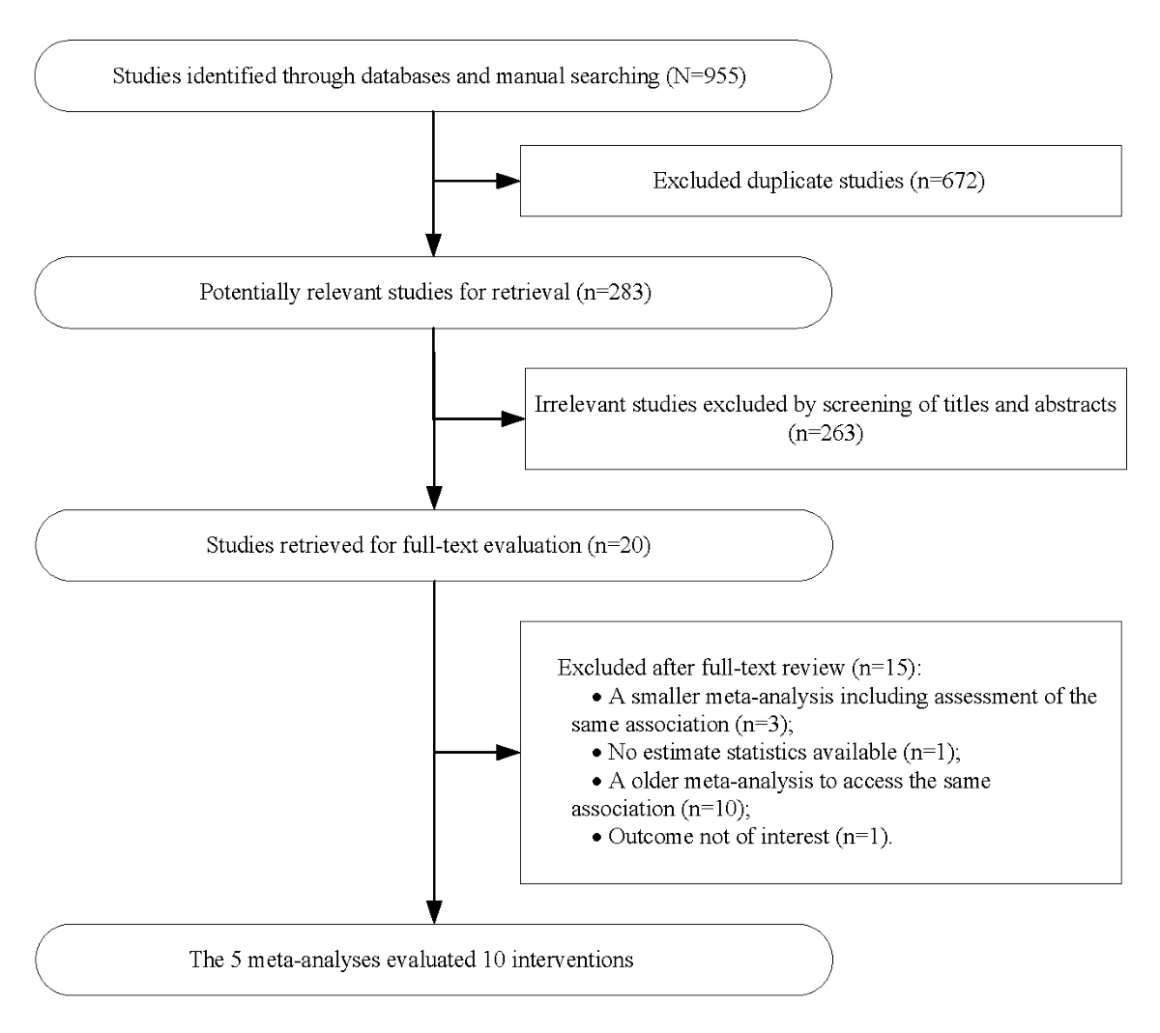
Flowchart of the study selection process.

We retrieved 284 meta-analyses from PubMed, 277 from Embase, 319 from Web of Science, and 75 from Cochrane. Two investigators independently screened the studies, and 5 meta-analyses were ultimately included in this umbrella review through 2 rounds of literature screening (Figure 1).

The number of studies included in the 5 meta-analyses ranged from 11 to 59, with a median of 7 (IQR 6-11). The sample size ranged from 580 to 4656, with a median of 407 (IQR 107-1490). The original research covered multiple regions, including Asia, Europe, Oceania, and the Americas. AMSTAR 2 conducted a methodological quality assessment of the 5 meta-analyses included in this umbrella review. Among them, 4 studies were rated as “high,” and 1 study was rated as “low” (Table 1).

**Table 1:** Characteristics and quality assessment of included meta-analyses to access interventions of digital addiction.

Author (year)	Regions of the original study	Study design	Studies (participants)	Population	Exposure	Follow-up	Comparison	Outcomes (type of ES^a^)	SE (95% CI)	AMSTAR 2^b^
Yeun (2016) [18]	East Asia	NRCT^c^ and RCT^d^	37 (1490)	IA^e^	Psychosocial intervention	NR^f^	Control	Improving self-control (SMD^g^) Self-esteem (MD^h^)	0.29 (0.11-0.47) 3.58 (2.03-5.12)	High
Stevens (2018) [22]	China, South Korea, Japan, Germany, United States, and Brazil	NR	12 (580)	IGD^i^	CBT^j^	3-6 months	Control	Reducing IGD symptoms (Hedges *g*) Depression (Hedges *g*) Anxiety (Hedges *g*)	0.92 (0.5-1.34) 0.8 (0.21-1.38) 0.55 (0.17-0.93)	High
Martin (2021) [42]	United States, Canada, Australia, Switzerland, Norway, New Zealand, and Japan	RCT, quasi-experimental	11 (4656)	Children	Interventions to reduce screen time in children	NR	Control	Screen use time (MD)	-0.56 (-0.92 to -0.2)	Low
Zhang (2022) [26]	China, Korea, and Iran	RCT	59 (3832)	IA	CBT, group counseling, sports intervention, and internet-based intervention	1 week to 5 months	No-treatment control group	Reducing the total scores of IA (SMD)	–1.9 (–2.26 to –1.55)	High
Zhang (2023) [21]	China	RCT	39 (2408)	IA student	Exercises	8-24 months	NR	Loneliness (SMD) Anxiety (SMD) Depression (SMD) Interpersonal sensitivity (SMD)	–0.96 (–1.5 to –0.41) –1.79 (–2.37 to –1.22) –1.5 (–2.19 to –0.81) –1.34 (–2.05 to –0.64)	High

^a^ES: effect statistics.

^b^AMSTAR 2: assessment of multiple systematic reviews version 2.

^c^NRCT: nonrandomized controlled trial.

^d^RCT: randomized controlled trial.

^e^IA: internet addiction.

^f^NR: not reported.

^g^SMD: standardized mean difference.

^h^MD: mean difference.

^i^IGD: internet gaming disorder.

^j^CBT: cognitive behavioral therapy.

### Quantitative Synthesis and Evidence Grading of the Meta-Analysis

Five meta-analyses evaluated 9 interventions: CBT, group counseling, exercise, integrated IA prevention programs, psychosocial interventions, reality therapy, self-control training programs, and interventions to reduce screen time in child and art therapy. The general characteristics of the studies included in the meta-analysis are presented in Table 1. Four of the selected meta-analyses were graded as “high quality” on the basis of the AMSTAR 2 score, and only 1 review was graded as “low quality.” However, the evidence class of most meta-analyses included evidence rated as “weak evidence” (19/21, 90%) and “not significant” (2/21, 10%). A summary of the quality appraisals of the meta-analyses and the AMSTAR 2 scores are presented in Table 1 and Multimedia Appendix 2 [18,21,22,26,42].

### Cognitive Behavioral Therapy

**Figure 2:**
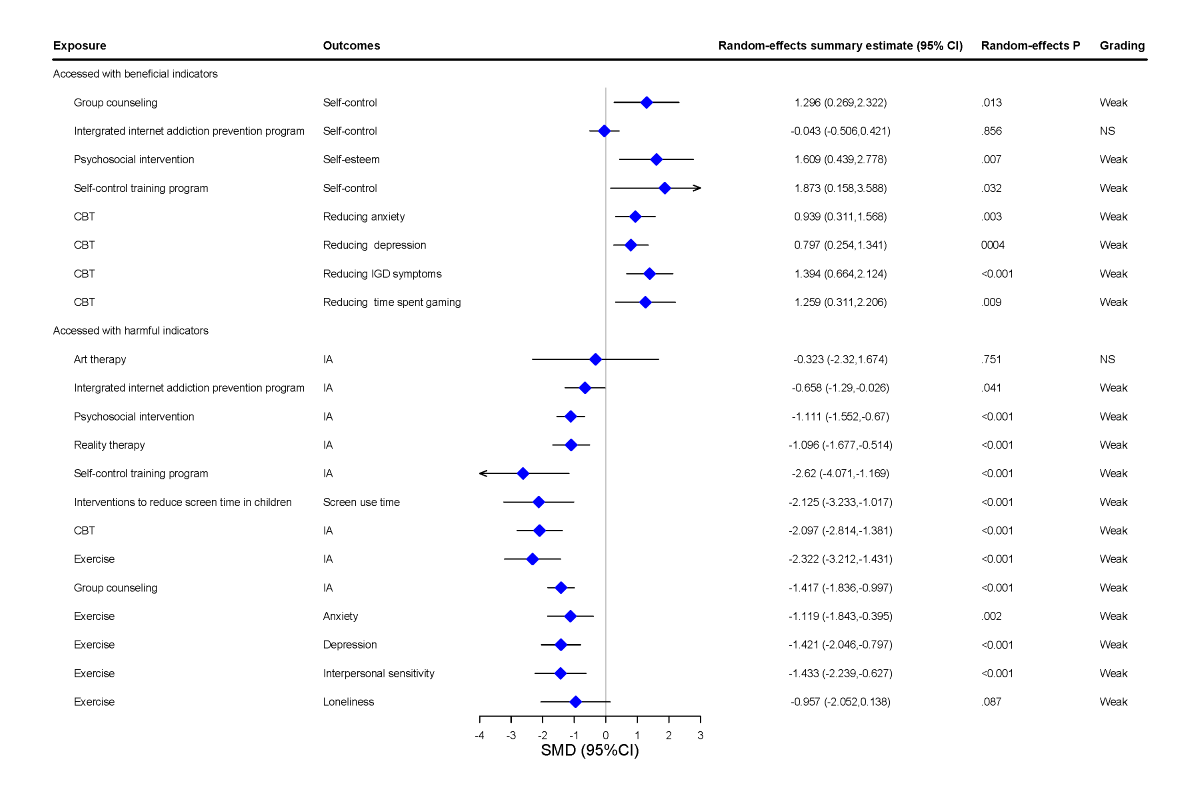
Forest plot of interventions for digital addiction. CBT: cognitive behavioral therapy; IA: internet addiction; IGD: internet gaming disorder; NS: not significant.

CBT is effective in treating substance abuse, gambling, and affective and eating disorders. CBTs are based on the cognitive behavioral model, which holds that thoughts determine feelings; thus, changing one’s thoughts can help with behavioral change [43]. CBT can reduce anxiety, depression, IGD symptoms, time spent gaming, and IA scores, as shown in this review. Two meta-analyses previously examined the effectiveness of CBT for patients with digital addiction [22,44]. For example, Stevens et al [22] report that CBT demonstrated high efficacy in reducing IGD symptoms (SMD 1.394, 95% CI 0.664-2.214) and depression (SMD 0.797, 95% CI 0.254-1.341) and moderate efficacy in reducing anxiety (SMD 0.939, 95% CI 0.311-1.586) and time spent on gaming (SMD 1.259, 95% CI 0.311-2.206). However, owing to the evidence base, there is a need for more rigorous studies to determine the potential long-term benefits of CBT for IGD [22]. The heterogeneity in the effects of CBT on reducing anxiety (*I*^2^=92.5), depression (*I*^2^=87.1), IGD symptoms (*I*^2^=96.6), and time spent gaming (*I*^2^=95.9) was high, although the effects of CBT on depression exhibited publication bias, albeit the evidence was weak. Another review explored the impact of CBT on IA levels [44] and found CBT to be significantly superior to routine intervention and paroxetine combined with buspirone drug therapy (*P* value of less than .05) on the IA test scale and also superior to routine intervention and no intervention (*P* value of less than .05) on the basis of the Revised Chen Internet Addiction scale and Young Diagnostic Questionnaire scale (*P* value of less than .05) [40]. Finally, the heterogeneity for the effects of CBT on reducing IA scores was high (*I*^2^=92.5; Figure 2 and Table 2).

**Table 2:** Quantitative synthesis and evidence grading of meta-analyses to access interventions of digital addiction.

Study (year)	Exposures	Comparison	Outcomes	Studies (participants)	Original effect metrics	Random-effects summary estimate (95% CI)	Random effects, *P* value	*I*^2^ (%)	95% prediction interval	Egger test>*P* value	Largest study estimate (95% CI)	Significant studies		Grading
												O/E^a^	*P* value	
Yeun and Han 2016) [18]	Art therapy	Control	IA^b^	3 (52)	SMD^c^	–0.323 (–2.32 to 1.674)	.751	90	(<0.001, >1000)	0.172	18.432 (2.909-114.686)	2/2.62	—^d^	NS^e^
Yeun and Han (2016) [18]	Group counseling	Control	Self-control	3 (70)	SMD	1.296 (0.269-2.322)	.013	66.9	(<0.001, >1000)	0.154	2.806 (0.865-9.266)	2/0.78	.17	Weak
Yeun and Han (2016) [18]	Integrated IA prevention program	Control	IA	4 (461)	SMD	–0.658 (–1.29 to –0.026)	.041	88.1	(0.002, 57.896)	0.027	1.095 (0.722-1.690)	3/0.23	<.01	Weak
Yeun and Han (2016) [18]	Integrated IA prevention program	Control	Self-control	4 (291)	SMD	–0.043 (–0.506 to 0.421)	.856	73.4	(0.025, 34.189)	0.319	0.320 (0.164-0.636)	1/2.64	—	NS
Yeun and Han (2016) [18]	Psychosocial intervention	Control	IA	16 (625)	SMD	–1.111 (–1.552 to –0.67)	<.001	82.5	(0.006, 3.123)	0.004	1.135 (0.581-2.178)	13/0.88	<.01	Weak
Yeun and Han (2016) [18]	Psychosocial intervention	Control	Self-esteem	6 (142)	MD^e^	1.609 (0.439-2.778)	.007	87.7	(0.010, >1000)	0.359	11.064 (2.490-49.162)	4/5.08	—	Weak
Yeun and Han (2016) [18]	Reality therapy	Control	IA	2 (54)	SMD	–1.096 (–1.677 to –0.514)	<.001	0	—	—	0.182 (0.050-0.672)	2/1.28	.54	Weak
Yeun and Han (2016) [18]	Self-control training program	Control	IA	5 (107)	SMD	–2.62 (–4.071 to –1.169)	<.001	85.6	(<0.001, 140.7)	0.020	0.503 (0.100-2.517)	4/0.67	<.01	Weak
Yeun and Han (2016) [18]	Self-control training program	Control	Self-control	3 (67)	SMD	1.873 (0.158, 3.588)	.032	86.8	(<0.001, >1000)	0.202	1.919 (0.435, 8.618)	2/0.39	.05	Weak
Stevens et al (2018) [22]	CBT^g^	Control	Reducing anxiety	7 (586)	Hedges *g*	0.939 (0.311-1.568)	.003	92.5	(0.097, 310.259)	0.216	2.34 (1.288-4.255)	4/3.34	.72	Weak
Stevens et al (2018) [22]	CBT	Control	Reducing depression	6 (303)	Hedges *g*	0.797 (0.254-1.341)	.004	87.1	(0.139, 128.859)	0.935	1.135 (0.592-2.178)	4/0.34	<.01	Weak
Stevens et al (2018) [22]	CBT	Control	Reducing IGD^h^ symptoms	11 (1164)	Hedges *g*	1.394 (0.664-2.124)	<.001	96.6	(0.072, >1000)	0.833	8.618 (6.222-11.938)	9/10.63	—	Weak
Stevens et al (2018) [22]	CBT	Control	Reducing time spent gaming	4 (397)	Hedges *g*	1.259 (0.311-2.206)	.009	95.9	(0.003, >1000)	0.226	74.277 (44.746-123.298)	4/4	≥.99	Weak
Martin et al (2021) [42]	Interventions to reduce screen time in children	Control	Screen use time	7 (1905)	MD	–2.125 (–3.233 to –1.017)	<.001	98.7	(<0.001, 34)	0.698	0.020 (0.014-0.029)	45450	—	Weak
Zhang et al (2022) [26]	CBT	No intervention	Total scores of IA	7 (491)	SMD	–2.097 (–2.814 to –1.381)	<.001	90.7	(<0.001, 2.094)	0.239	0.048 (0.022-0.102)	7/6.96	≥.99	Weak
Zhang et al (2022) [26]	Exercise	No intervention	Total scores of IA	4 (285)	SMD	–2.322 (–3.212 to –1.431)	<.001	88.1	(<0.001, 27.397)	0.247	0.031 (0.012, 0.079)	4/4	≥.99	Weak
Zhang et al (2022) [26]	Group counseling	No intervention	Total scores of IA	11 (416)	SMD	–1.417 (–1.836 to –0.997)	<.001	70.3	(0.006, 0.978)	0.545	0.215 (0.082-0.550)	10/7.18	.11	Weak
Zhang et al (2023) [21]	Exercise	Control	Anxiety	9 (362)	SMD	–1.119 (–1.843 to –0.395)	.002	87	(0.003, 7.315)	0.492	0.207 (0.071-0.613)	6/6.24	—	Weak
Zhang et al (2023) [21]	Exercise	Control	Depression	4 (102)	SMD	–1.421 (–2.046 to –0.797)	<.001	70.7	(0.001, 9.428)	0.073	0.176 (0.104-0.625)	4/2.45	.16	Weak
Zhang et al (2023) [21]	Exercise	Control	Interpersonal sensitivity	5 (144)	SMD	–1.433 (–2.239 to –0.627)	<.001	79.9	(<0.001, 14.804)	0.106	0.819 (0.215-3.128)	4/0.3	<.01	Weak
Zhang et al (2023) [21]	Exercise	Control	Loneliness	8 (416)	SMD	–0.957 (–2.052 to 0.138)	.087	93.1	(<0.001, 5.319)	0.841	0.108 (0.034-0.350)	6/7.16	—	Weak

^a^O/E: Observed number of studies with positive findings/expected number of studies with positive findings.

^b^IA: internet addiction.

^c^SMD: standardized mean difference.

^d^—: not applicable.

^e^NS: not significant.

^f^MD: mean difference.

^g^CBT: cognitive behavioral therapy.

^h^IGD: internet gaming disorder.

### Group Counseling

The main components of group counseling include identifying previous solutions and acknowledging problems while also realizing that exceptions to the problem are key to the solution, with a focus on the present and future [45]. Two meta-analyses previously explored the effectiveness of group counseling on digital addiction. Yeun et al [18] reported that group counseling had a large effect on improving self-control among school-aged children with IA (SMD 1.296, 95% CI 0.269-2.322). The heterogeneity for the effects of group counseling on improving self-control was high (*I*^2^=66.9), but the evidence was weak. Another review reported that group counseling reduced IA levels (SMD –1.417, 95% CI –1.836 to –0.997) [44]. The heterogeneity for the effects of group counseling on reducing IA scores was high (*I*^2^=95.9; Figure 2 and Table 2).

### Exercises

Exercise is an important intervention method that positively impacts individual cognition, emotion, and physical fitness [5]. Two meta-analyses examined this intervention’s effectiveness in reducing digital addiction outcomes. One review reported that sports interventions reduced IA scores (SMD –2.322, 95% CI –3.212 to –1.431) [44]. The heterogeneity for the effects of exercise on reducing IA scores was high (*I*^2^=88.1), although the evidence was weak. Another review conducted by Zhang et al [21] included a total of 2408 students from 39 RCTs. Six exercise types (team sport, double sport, single sport, team + double sport, team + single sport, and team + double + single sport) were compared on the basis of their effectiveness in reducing IA and maintaining mental health. The meta-analysis results revealed that, compared with the control group, exercising significantly improved loneliness, anxiety, depression, and interpersonal sensitivity (*P* value of less than .05) [21]. The heterogeneity in the effects of exercise on improving loneliness (*I*^2^=93.1), anxiety (*I*^2^=87), depression (*I*^2^=70.7), and interpersonal sensitivity (*I*^2^=79.9) was high (*I*^2^=95.9; Figure 2 and Table 2).

### Psychosocial Interventions

Psychosocial interventions capitalize on psychological or social actions to produce changes in psychological, social, biological, and functional outcomes [46]. One review examined the efficacy of psychosocial interventions on school-aged children’s IA, self-control, and self-esteem and revealed that psychosocial interventions had a strong effect on reducing IA (SMD –1.111, 95% CI –1.552 to –0.67) and improving self-esteem (SMD 1.609, 95% CI 0.439-2.778) [18]. The effect on IA had high heterogeneity (*I*^2^=82.5) and published bias (*P*=.004), and the heterogeneity of self-esteem was high (*I*^2^=87.7; Figure 2 and Table 2).

### Integrated IA Prevention Program

The integrated IA prevention (IIAP) program combines 2 or more interventions, including CBT, play, art therapy, conflict, stress management, and CBT. [18] A meta-analysis of data on the efficacy of IIAP for self-control and IA revealed that IIAP had no effect on self-control (SMD –0.043, 95% CI –0.506 to 0.421), although the evidence was insignificant [18]. IIAP was found to reduce the IA, and while the evidence was weak, the effect showed high heterogeneity (*I*^2^=88.1), publication bias (*P=.*027), and excessive significance (observed number of studies with positive findings=3; expected number of studies with positive findings=0.23; *P* value of less than .01; Figure 2 and Table 2).

### Self-Control Training Program

A meta-analysis of data on the efficacy of self-control training programs for self-control and IA reported in 5 studies revealed that self-control training programs improved self-control (SMD 1.873, 95% CI 0.158 to 3.588) and reduced IA (SMD –2.62, 95% CI –4.071 to –1.169) [18], but the evidence was weak. In addition, the effects were highly heterogeneous and highly significant (*P* value of less than .1). There was a publication bias in terms of the effect on IA (*P=.*02; Figure 2 and Table 2).

### Reality Therapy

Weak evidence has indicated that in a review of the effect of reality therapy on IA based on two trials, reality therapy reduced IA (SMD –1.096, 95% CI –1.677 to 0.514) [18] without heterogeneity between the 2 trials (Figure 2 and Table 2).

### Interventions to Reduce Screen Time in Children

A meta-analysis suggested that interventions for screen use and sleep can achieve a small reduction in screen time (SMD –2.125, 95% CI –3.233 to –1.017) [47]. Heterogeneity was high (*I*^2^=98.7), and the evidence was weak (Figure 2 and Table 2).

## Discussion

### Principal Findings

This umbrella review provides an overview of the relationships between digital addiction intervention-related outcome indicators and intervention methods derived from 5 meta-analyses. Four of the selected meta-analyses were of “high” quality. We found that most interventions (eg, CBT and exercise) can improve related outcomes, such as lifestyle, emotional health, mental health, and social relationships, among people with digital addiction. However, this conclusion was derived from weak evidence and has significant heterogeneity and excessive significance. The evidence in this review was rated as “weak evidence” (19/21, 90%) or “not significant” (2/21, 10%). The evidence was downgraded to “weak” considering that most of the included studies were RCTs, which generally involved a limited number of participants, that is, fewer than 1000 in total after pooled analyses, easily causing the small sample sizes limitation, and second, the articles were considered to have exhibited heterogeneity as well as publication bias, suggesting that more high-quality, credible research is needed to further clarify the associations between intervention methods and digital addiction outcomes.

### Comparison With Prior Work

On the basis of the results of this umbrella review, CBT not only reduces IA scores but also reduces IGD symptoms, depression, and anxiety. CBT, the psychotherapy most commonly used for digital addiction, can improve symptoms of depression and anxiety by reshaping negative thoughts to help patients understand routine and problematic behaviors and motivate the creation of more adaptive, goal-oriented routines [48]. Compared with other interventions (such as drug replacement therapy), CBT has the advantage of being less invasive and effective in the short term. Furthermore, CBT is the most effective intervention for reducing internet use and improving self-perception [49]. CBT emphasizes the connection among behaviors, thoughts, and emotions, prompting patients to pay attention to these behaviors [20]. A previous meta-analysis revealed that CBT was associated with positive changes in depression, anxiety, and psychoticism in patients with IA [20]. Ding KY et al [48] also reported that most interventions for digital addiction in children and adolescents were CBT- or CBT-based interventions, which can improve anxiety, depression, and digital addiction-related symptoms. However, another meta-analysis did not conclude that the CBT intervention had a significant effect on the severity of IA [50]. Our analysis of the effect of CBT on digital addiction suggested that genuine heterogeneity likely exists. The high heterogeneity might be related not only to the potential bias in the original studies but also to the differences across the studies included in this meta-analysis. These associations are both supported by a weak level of evidence; thus, more studies are needed to further document the effect of CBT on patients with digital addiction.

There is evidence that group counseling is one of the main treatments for addiction. One of the advantages of group programs is their economic value since they target many students simultaneously [51]. A previous study reported that groups of fewer than 14 people presented effect sizes exceeding 90% [52]. Group counseling programs enable group members to understand and help each other, increase social participation and interpersonal interactions among patients, improve adaptability, develop personalities, and eliminate the symptoms of digital addiction [53]. Group counseling programs can gain patient support and insight through experiencing similar cognitions and emotions, providing a major modality in the treatment of digital addiction [54]. In this review, we conclude that group counseling improved self-control and reduced IA scores among patients with digital addiction. A meta-analysis suggested that group counseling programs improve relationships, health problems, time management, tolerance, and compulsive internet use [20]. A previous meta-analysis by Park et al [55] identified the beneficial effects of group counseling programs for adolescents with IA. However, group counseling may not be truly beneficial until the barriers related to social anxiety, social isolation, and a lack of social skills are overcome. Therefore, developing a protocol for appropriate methods on the basis of the characteristics of the participants is necessary.

Exercise, including running, badminton, basketball, table tennis, and other outdoor sports games, was found to be helpful in reducing IA scores and improving loneliness, anxiety, depression, and interpersonal sensitivity in patients with digital addiction. Exercise is an important intervention method that positively impacts individual cognition, emotion, and physical fitness, in addition to psychological interventions. Digital addiction alters the neural structure, reduces the activity of the dopaminergic system, and limits neurocognitive functions [56]. However, exercise-based interventions can effectively improve the autonomic nervous system in patients with IA and normalize, to a certain extent, the structure of specific parts of the central nervous system [57]. Moreover, it can increase plasma glial cell-derived neurotrophic factor and glucocorticoid levels [58], neurotransmitter release [57], and telomere protection [59]. Exercise-based interventions have been shown to reduce the prevalence and symptoms of IA, and participation in physical activity may prevent IA. Exercise has replaced most internet experiences and improved an individual’s physical and mental health [19]. Long-term physical exercise has been found to significantly reduce the degree of IA and depression and improve sleep quality among college students with IA [44]. The meta-analysis included in this review of exercise interventions included only exercise type classification, that is, it did not compare exercise intensity or exercise strength. Thus, more studies on exercise interventions are needed to demonstrate their effects on digital addiction.

Digital addiction may constitute impulsive behavior, although the consequences are negative impacts and involve continuous participation [60]. The impulse theory suggests that the behavior of addiction is the result of the generally excessive active brain reward system. Individuals with excessively active brain reward systems may show strong reactions to the prompts of predicting potential rewards, thereby explaining the pursuit of novelty, impulses, and powerful motivation to obtain internet or other addictive stimuli [61]. Therefore, impulses have been proposed as potential marks and treatment targets of digital addiction [62]. Reality therapy helps individuals control their behavior and consider other options related to internet use [63]. As reality therapy is based on the theory of selecting, the theory holds that people are responsible for their own lives, their feelings, their actions, and their behaviors [64]. In a sense, reality therapy helps individuals reflect on their behavior, evaluate their choices, choose more effective choices, and directly target the choice and self-control of target guidance [64]. In this method, the participants are required to determine the goals of their behavior and realize what they are actually doing. They were then guided to determine whether their behavior promoted or hindered their ability to achieve their original goals. Finally, they are encouraged to seek more appropriate and healthier alternatives to replace their current behavior, achieve their goals, and formulate plans to change negative behaviors [63]. Real-world therapy has been widely used to treat addictive diseases, including diseases involving drugs, sex, and food [63]. For people with addiction and impulses, reality therapy can help them improve their self-control and thereby reduce their problems. Research has shown that reality therapy can increase self-esteem and decrease IA [64]. Similar impacts have also been proven in the study of material addiction in real-world therapy [65]. In this review, we found that real-world therapy reduces IA scores. Despite the study being graded as having high methodological quality, the level of research evidence was weak due to the small number of participants included in the study.

IIAPs are characterized by the simultaneous or sequential delivery of several different types of interventions, including pharmacology, psychotherapy, and group counseling. A composite approach may help solve complex problems, whereas combinations of different therapies with different therapeutic targets and mechanisms may produce synergistic effects. The effects of CBT in combination with other psychosocial or pharmacological therapies for IA have been studied and determined that the combination of CBT with other therapies affects IA severity, somatization, paranoid ideation, psychosis, obsessive-compulsive traits, interpersonal sensitivity, depression, and anxiety [66]. In this umbrella review, the assessments of IIAPs had high between-study heterogeneity, with public bias and excessive salience, and the evidence of the effect on IA scores was weak. The IIAP showed a null evidence class of effects on self-control, suggesting that there is no clear scientific evidence to support the link between IIAP and self-control in patients with digital addiction.

The evidence reviewed here suggests that CBT, group counseling, exercise, IIAP programs, psychosocial interventions, reality therapy, self-control training programs, and interventions to reduce screen time may be beneficial to patients with digital addiction. Nonetheless, more robust population-based studies are essential to substantiate these potential benefits, as the current evidence is largely inconclusive or weak. It is imperative to conduct further research to evaluate the long-term efficacy and cost-effectiveness of these interventions, as well as to investigate their suitability and generalizability across diverse cultural and socioeconomic settings.

### Limitations

This umbrella review has certain limitations. First, this review was based on the results of published and available meta-analyses and meta-analyses, so other results that have not been evaluated by meta-analysis and missing data in the original literature could not be considered. However, our results were not significantly affected because repeated meta-analytic evaluations yielded similar results. Second, throughout the study, original studies covering multiple regions were included in this analysis, and although we were not able to describe subgroups of regions based on the meta-analysis that was conducted, a cross-cultural analysis of multiple geographic sources provides a better description of the effects of various interventions on digital addiction. Third, studies suggest that gender plays a role in digital addiction, as evidenced by the greater susceptibility of males, but the meta-analysis included did not stratify gender to examine the differences in the efficacy of interventions for digital addiction and therefore cannot be discussed further in this study [67]. Fourth, the statistical methods we used to test for the presence of bias indicate only its existence, not its exact source. Finally, the methodological quality of the meta-analyses included in this umbrella review and the quality of evidence for most prognosis-related outcome indicators were not sufficient. Therefore, more high-quality studies are needed in the future to further verify the results of this study.

## Appendix

Multimedia Appendix 1 Excluded studies with reasons for exclusion.

Multimedia Appendix 2 Result of the Assessment of Multiple Systematic Reviews version 2 (AMSTAR-2).

## Abbreviations

AMSTAR 2: Assessment of Multiple Systematic Reviews version 2
CBT: cognitive behavioral therapy
GRADE: Grading of Recommendations, Assessment, Development, and Evaluations
IA: internet addiction
IGD: internet gaming disorder
IIAP: integrated internet addiction prevention
MD: mean difference
PRISMA: Preferred Reporting Items for Systematic Reviews and Meta-Analyses
RCT: randomized controlled trial
SMD: standardized mean difference
