# Supplementary material for: Interventions for Digital Addiction: Umbrella Review of Meta-Analyses
Source: J Med Internet Res. 2025 Feb 11;27:e59656. doi: 10.2196/59656 (PMC11862776; doi:10.2196/59656)
Supplement: Multimedia Appendix 1 [file jmir_v27i1e59656_app1.docx]

**Table S1. Excluded studies with reasons for exclusion.**

| NO. | Excluded studies | Title | Reasons for exclusion | Studies that evaluated same associations and were finally included in umbrella review |
| --- | --- | --- | --- | --- |
| 1 | Kim 2022[1] | Psychological treatments for excessive gaming: a systematic review and meta-analysis | Similar meta-analysis with a with a smaller sample included | Zhang 2022[2] |
| 2 | Chang 2022[3] | The Comparative Efficacy of Treatments for Children and Young Adults with Internet Addiction/Internet Gaming Disorder: An Updated Meta-Analysis | Similar meta-analysis with a with a smaller sample included | Yeun 2016[4] |
| 3 | Andrade 2022[5] | The Effect of Psychosocial Interventions for Reducing Co-occurring Symptoms of Depression and Anxiety in Individuals with Problematic Internet Use: A Systematic Review and Meta-analysis | Similar meta-analysis with a with a smaller sample included | Yeun 2016[4] |
| 4 | Lu 2021[6] | The correlation between mobile phone addiction and coping style among Chinese adolescents: a meta-analysis | No complete data available | NA |
| 5 | Goslar 2020[7] | Treatments for internet addiction, sex addiction and compulsive buying: A meta-analysis | Similar meta-analysis with a with a smaller sample included | Zhang 2022[2] |
| 6 | Liu 2019[8] | Exercise as an Alternative Approach for Treating Smartphone Addiction: A Systematic Review and Meta-Analysis of Random Controlled Trials | Similar meta-analysis with a with a older sample included | Zhang 2023[9] |
| 7 | Liu 2017[10] | Effects of Group Counseling Programs, Cognitive Behavioral Therapy, and Sports Intervention on Internet Addiction in East Asia: A Systematic Review and Meta-Analysis | Similar meta-analysis with a with a older sample included | Zhang 2022[2] |
| 8 | Chun 2017[11] | A Meta-Analysis of Treatment Interventions for Internet Addiction Among Korean Adolescents | Similar meta-analysis with a with a older sample included | Zhang 2022[2] |
| 9 | Augner 2022[12] | Tackling the 'digital pandemic': The effectiveness of psychological intervention strategies in problematic Internet and smartphone use-A meta-analysis | Similar meta-analysis with a with a smaller sample included | Zhang 2022[2] |
| 10 | Augner 2022[13] | Psychological online interventions for problem gambling and gambling disorder - A meta-analytic approach | Non-interested outcome | NA |
| 11 | Winkler 2013[14] | Treatment of internet addiction: a meta-analysis | No complete data available | NA |
| 12 | Danielsen 202[15] | Treatment effects of therapeutic interventions for gaming disorder: A systematic review and meta-analysis | No complete data available | NA |
| 13 | Jiang 2023[16] | Effects of non-pharmacological interventions on youth with internet addiction: a systematic review and meta-analysis of randomized controlled trials | Similar meta-analysis with a with a smaller sample included | Yeun 2016[4] |
| 14 | Li 2023[17] | Exercise intervention to reduce mobile phone addiction in adolescents: a systematic review and meta-analysis of randomized controlled trials | Similar meta-analysis with a with a smaller sample included | Yeun 2016[4] |

## References:

1. Kim J, Lee S, Lee D, et al. Psychological treatments for excessive gaming: a systematic review and meta-analysis. Scientific reports. 2022 Nov 28;12(1):20485. doi: 10.1038/s41598-022-24523-9. PubMed PMID: 36443408; PubMed Central PMCID: PMCPMC9705304. eng.

2. Zhang X, Zhang J, Zhang K, et al. Effects of different interventions on internet addiction: A meta-analysis of random controlled trials. Journal of affective disorders. 2022 Sep 15;313:56-71. doi: 10.1016/j.jad.2022.06.013. PubMed PMID: 35760188; eng.

3. Chang CH, Chang YC, Yang L, et al. The Comparative Efficacy of Treatments for Children and Young Adults with Internet Addiction/Internet Gaming Disorder: An Updated Meta-Analysis. International journal of environmental research and public health. 2022 Feb 24;19(5). doi: 10.3390/ijerph19052612. PubMed PMID: 35270305; PubMed Central PMCID: PMCPMC8909504. eng.

4. Yeun YR, Han SJ. Effects of Psychosocial Interventions for School-aged Children's Internet Addiction, Self-control and Self-esteem: Meta-Analysis. Healthcare informatics research. 2016 Jul;22(3):217-30. doi: 10.4258/hir.2016.22.3.217. PubMed PMID: 27525163; PubMed Central PMCID: PMCPMC4981582. eng.

5. Andrade ALM, Di Girolamo Martins G, Scatena A, et al. The Effect of Psychosocial Interventions for Reducing Co-occurring Symptoms of Depression and Anxiety in Individuals with Problematic Internet Use: A Systematic Review and Meta-analysis. International journal of mental health and addiction. 2022 Jun 3:1-22. doi: 10.1007/s11469-022-00846-6. PubMed PMID: 35677712; PubMed Central PMCID: PMCPMC9164571. eng.

6. Lu GL, Ding YM, Zhang YM, et al. The correlation between mobile phone addiction and coping style among Chinese adolescents: a meta-analysis. Child and adolescent psychiatry and mental health. 2021 Oct 15;15(1):60. doi: 10.1186/s13034-021-00413-2. PubMed PMID: 34654451; PubMed Central PMCID: PMCPMC8520246. eng.

7. Goslar M, Leibetseder M, Muench HM, et al. Treatments for internet addiction, sex addiction and compulsive buying: A meta-analysis. Journal of behavioral addictions. 2020 Apr 1;9(1):14-43. doi: 10.1556/2006.2020.00005. PubMed PMID: 32359229; PubMed Central PMCID: PMCPMC8935188. eng.

8. Liu S, Xiao T, Yang L, et al. Exercise as an Alternative Approach for Treating Smartphone Addiction: A Systematic Review and Meta-Analysis of Random Controlled Trials. International journal of environmental research and public health. 2019 Oct 15;16(20). doi: 10.3390/ijerph16203912. PubMed PMID: 31618879; PubMed Central PMCID: PMCPMC6843500. eng.

9. Zhang Y, Li G, Liu C, et al. Mixed comparison of interventions for different exercise types on students with Internet addiction: a network meta-analysis. Frontiers in psychology. 2023;14:1111195. doi: 10.3389/fpsyg.2023.1111195. PubMed PMID: 37303910; PubMed Central PMCID: PMCPMC10249056. eng.

10. Liu J, Nie J, Wang Y. Effects of Group Counseling Programs, Cognitive Behavioral Therapy, and Sports Intervention on Internet Addiction in East Asia: A Systematic Review and Meta-Analysis. International journal of environmental research and public health. 2017 Nov 28;14(12). doi: 10.3390/ijerph14121470. PubMed PMID: 29182549; PubMed Central PMCID: PMCPMC5750889. eng.

11. Chun J, Shim H, Kim S. A Meta-Analysis of Treatment Interventions for Internet Addiction Among Korean Adolescents. Cyberpsychology, behavior and social networking. 2017 Apr;20(4):225-231. doi: 10.1089/cyber.2016.0188. PubMed PMID: 28263660; eng.

12. Augner C, Vlasak T, Aichhorn W, et al. Tackling the 'digital pandemic': The effectiveness of psychological intervention strategies in problematic Internet and smartphone use-A meta-analysis. The Australian and New Zealand journal of psychiatry. 2022 Mar;56(3):219-229. doi: 10.1177/00048674211042793. PubMed PMID: 34477009; eng.

13. Augner C, Vlasak T, Aichhorn W, et al. Psychological online interventions for problem gambling and gambling disorder - A meta-analytic approach. Journal of psychiatric research. 2022 Jul;151:86-94. doi: 10.1016/j.jpsychires.2022.04.006. PubMed PMID: 35472684; eng.

14. Winkler A, Dörsing B, Rief W, et al. Treatment of internet addiction: a meta-analysis. Clinical psychology review. 2013 Mar;33(2):317-29. doi: 10.1016/j.cpr.2012.12.005. PubMed PMID: 23354007; eng.

15. Danielsen PA, Mentzoni RA, Lag T. Treatment effects of therapeutic interventions for gaming disorder: A systematic review and meta-analysis. Addictive behaviors. 2024 Feb;149:107887. doi: 10.1016/j.addbeh.2023.107887. PubMed PMID: 37826910.

16. Jiang YS, Liu TH, Qin D, et al. Effects of non-pharmacological interventions on youth with internet addiction: a systematic review and meta-analysis of randomized controlled trials. Frontiers in psychiatry. 2023;14:1327200. doi: 10.3389/fpsyt.2023.1327200. PubMed PMID: 38274427; PubMed Central PMCID: PMCPMC10808612.

17. Li Z, Xia X, Sun Q, et al. Exercise intervention to reduce mobile phone addiction in adolescents: a systematic review and meta-analysis of randomized controlled trials. Frontiers in psychology. 2023;14:1294116. doi: 10.3389/fpsyg.2023.1294116. PubMed PMID: 38192396; PubMed Central PMCID: PMCPMC10773895.
